# Analyzing Psychotherapy on Twitter: An 11-Year Analysis of Tweets From Major U.S. Media Outlets

**DOI:** 10.3389/fpsyt.2022.871113

**Published:** 2022-05-18

**Authors:** Miguel A. Alvarez-Mon, Cesar Ignacio Fernandez-Lazaro, Miguel A. Ortega, Cristina Vidal, Rosa M. Molina-Ruiz, Melchor Alvarez-Mon, Miguel A. Martínez-González

**Affiliations:** ^1^Department of Medicine and Medical Specialities, Faculty of Medicine and Health Sciences, University of Alcala, Alcala de Henares, Spain; ^2^Ramón y Cajal Institute of Sanitary Research (IRYCIS), Madrid, Spain; ^3^Department of Psychiatry and Mental Health, Hospital Universitario Infanta Leonor, Madrid, Spain; ^4^Department of Preventive Medicine and Public Health, School of Medicine, University of Navarra, Pamplona, Spain; ^5^Navarra's Health Research Institute (IdiSNA), Pamplona, Spain; ^6^Department of Psychiatry and Medical Psychology. University of Navarra Clinic, Pamplona, Spain; ^7^Department of Psychiatry and Mental Health, Hospital Universitario Clínico San Carlos, Madrid, Spain; ^8^Centro de Investigación Biomédica en Red Fisiopatología de la Obesidad y Nutrición (CIBERobn), Institute of Health Carlos III, Madrid, Spain

**Keywords:** Twitter, psychotherapy, public health, health promotion, health communication

## Abstract

**Background:**

The Internet has become the main source of information on health issues, and information now determines the therapeutic preferences of patients. For this reason, it is relevant to analyze online information discussing psychotherapy.

**Objective:**

To investigate tweets posted by 25 major US media outlets between 2009 and 2019 concerning psychotherapy.

**Methods:**

We investigated tweets posted by 25 major US media outlets about psychotherapy between January 2009 and December 2019 as well as the likes generated. In addition, we measured the sentiment analysis of these tweets.

**Results:**

Most of the tweets analyzed focused on Mindfulness (5,498), while a low number were related to Psychoanalysis (376) and even less to Cognitive-Behavioral Therapy (61). Surprisingly, Computer-supported therapy, Psychodynamic therapy, Systemic therapy, Acceptance and commitment therapy, and Dialectical behavior therapy did not generate any tweet. In terms of content, efficacy was the main focus of the posted tweets, receiving Cognitive-Behavioral Therapy and Mindfulness a positive appraisal.

**Conclusions:**

US media outlets focused their interest on Mindfulness which may have contributed to the growing popularity in the past years of this therapeutic modality.

## Introduction

Mental disorders are a major problem in the world because they result in significant suffering and functional impairment (1). Their treatment is based on different approaches including psychotherapy and pharmacotherapy. In fact, several studies have found that the combination of both may be more effective than either of these alone (2, 3). Psychotherapy has been proven to be an effective intervention for multiple mental disorders (4). Specific psychotherapies are often individualized in patients or groups with a psychiatric disorder, or for those suffering from adverse circumstances. There are many types of psychotherapy with varying methods and levels of empirical support. The choice of the most appropriate type of psychotherapy is in part based upon a patient's specific problem or diagnosis and his/her preferences for treatment (5–7). For this reason, it is especially important that people potentially interested in psychotherapy have access to correct information.

Several factors modulate the efficacy of psychotherapy, but trust in the process is key (8). Patients' confidence in this therapeutic modality depends on many factors, including what they have read about treatment (9–11). Nowadays most sectors of the general population turn to the internet to search for medical information (12, 13). According to a Pew Research Center survey, roughly seven-in-ten Americans use social media, a level that has remained relatively stable over the past several years (14). In addition, most Americans who use social media, specifically Twitter, get news on this platform (15). In fact, the average U.S. adult Twitter user focused on health in 8% of their news-related tweets in 2021 (16). More importantly, in almost half of those tweets, users included their opinion about the news. From a public health perspective, it is very important to analyze this information because Twitter has emerged as a medical discussion forum (17–19). Additionally, media outlets have a great influence on forming reader opinions about the topics that are discussed in the news (20). Therefore, health news can ultimately affect patients' treatment choices.

In this context, infodemiology, an area of medical research focused on scanning the internet for user-contributed health-related content, has emerged as a means for understanding trends in public health (21, 22). Moreover, multiple studies have explored the interests and feelings of the general population regarding certain health problems through the analysis of social media posts (23–25). Nevertheless, there is a lack of data assessing social interest toward psychotherapy. We attempted to address this gap by examining the distribution of tweets on the topic of psychotherapy in a number of highly recognized, relevant U.S. media outlets. We selected psychotherapies with proven health benefits, including reduced symptomatology and the treatment of many chronic diseases, particularly anxiety and depression. Thus, we aimed to investigate tweets posted by 25 major U.S. media outlets between 2009 and 2019 concerning psychotherapy. In addition, we measured all generated likes as an indicator of Twitter users' interest in this topic.

## Methods

### Collection of Twitter Data

The focus of this study was centered on tweets, a short message of a maximum of 280 characters posted on Twitter, concerning psychotherapy posted by a sample of 25 major U.S. media outlets beginning in January 2009 and concluding in December 2019. A representative sample of different categories of media sites was selected, including 11 newspapers (The New York Times, Washington Post, Los Angeles Times, USA Today, The Chicago Tribune, New York Post, Wall Street Journal, New York Daily News, Boston Globe, San Francisco Chronicle, British Daily Mail), 6 broadcast network television or cable news sites (MSNBC, CNN, ABC News, Fox News, CBS News, BBC News), 1 wire service news site (Reuters), 3 hybrid online-only sites (Yahoo News, AOL News, Huffington Post) and 4 news aggregators (Google News, The Examiner, Topix, Bing News). These media outlets, which are among the most influential in the U.S., though the headquarters of some are based in other countries, were selected based on the number of Twitter followers, as shown by their individual accounts and their social influence during the course of the study (26).

### Search Strategy

The research strategy utilized focused on searching for tweets referring to psychotherapy. All tweets posted from the Twitter accounts of the previously mentioned media outlets were investigated and filtered according to specific criteria and the following list of keywords: 1. Computer-supported therapy; 2. Cognitive-behavioral therapy; 3. Psychoanalysis; 4. Psychodynamic therapy; 5. Systemic therapy; 6. Acceptance and commitment therapy; 7. Dialectical behavior therapy; 8. Mindfulness. The inclusion criteria for tweets were: (1) Tweets posted by any of the 25 U.S. media outlets selected for the study; (2) Use of the previously mentioned keywords; (3) Tweets posted between January 1st 2009 and December 31st 2019; (4) Text in English. The eleven-year period was chosen to align our research with the past decade. Lastly, those tweets that provided information that was too limited (i.e., tweets consisting mainly of hashtags) or contained only pictures were excluded.

### Search Tool Used

In this study, Twitter Firehose, handled by GNIP, was used, which allows access to 100% of all public tweets that match some sort of “search” criteria or query (27). In this study, the search criteria used was the previously indicated keywords. Tweet Binder, the search engine employed, uses node.js and PHP language, meaning it is able to analyze tweets in json format, which GNIP is then able to read.

### Content Analysis Process

First, all of the tweets were scanned, excluding those that provided information or included hashtags on more than one psychotherapy modality. All remaining tweets were considered for thematic content analysis. Secondly, a codebook was created based on our research questions, our previous experience in analyzing tweets, and what we determined to be the most common tweet themes (28–31). Third, two raters separately analyzed 100 tweets to test the suitability of the codebook. Discrepancies were discussed between these raters and with another rater. After revising the codebook, the three raters then proceeded to manually analyze the content of 5,935 tweets. First, they examined whether a tweet mentioned a particular psychotherapy as effective or non-effective with regards to general well-being. Secondly, they expanded on this data to see if each psychotherapy, whether effective or non-effective, was linked to a specific physical or mental health condition. Third, in those cases where a link was included within a tweet, the link was categorized as either scientific or non-scientific depending on its source. Scientific sources were considered those originating from academic institutions, hospitals and official websites.

### Measuring Interest and Sentiment on Twitter

We analyzed the number of likes generated by each tweet as an indicator of user interest in a given topic (32). In addition, the positivity or negativity of a hashtag was measured on a scale from 1 (negative) to 100 (positive) (33). Sentiment analysis tools evaluate all the words contained in each tweet, and each word has its own score that can vary depending on the context. In the case of this study, the average score of all the tweets with a certain hashtag determined the overall sentiment score. According to that score, each hashtag was classified into five categories: very negative (0–20), negative (>20–40), neutral (>40–60), positive (>60–80) and very positive (>80–100). Tweet Binder, the search engine that was used, has a service that uses a modified version of AFINN-165 (a list of English words rated for valence) to calculate a sentiment score for any given tweet.

### Ethical Considerations

This study received the approval of the University of Alcalá Research Ethics Committee and was compliant with the research ethics principles contained in the Declaration of Helsinki (7th revision, 2013). However, this study did not directly involve human subjects nor include any interventions but instead used publicly available tweets.

### Statistical Analysis

Descriptive statistics, including frequency and proportions, were computed to summarize tweets and their respective likes. The ratio of likes per tweet was calculated by dividing the number of likes by the number of tweets for each psychotherapy. Any differences in the number of tweets by month and year were determined by the Kruskal–Wallis H test.

## Results

### Overall Tweets, Likes, and Retweets

A total number of 5,935 tweets about psychotherapy were generated by 25 major U.S. media outlets between 2009 and 2019 (Table 1). The number of tweets about each of the analyzed psychotherapy modalities followed a heterogeneous pattern of distribution. In particular, mindfulness accounted for almost all of the tweets (92.6%). On the other hand, tweets related to psychoanalysis accounted for 6.3% of the total, and this was followed by those referencing cognitive-behavioral therapy (1%). Of note, the other five psychotherapy modalities included in our study (computer-supported therapy, psychodynamic therapy, systemic therapy, acceptance and commitment therapy, and dialectical behavior therapy) did not generate any tweets.

**Table 1:** Number of tweets posted from 2009 to 2019 by 25 major US media outlets about psychotherapies and the likes generated by Twitter users.

	**Tweets**		**Likes**		**Ratio likes/tweets**
	***n*** **(frequency)**	**% (percentage)**	***n*** **(frequency)**	**% (percentage)**	
CBT	61	1.0	153	1.2	2.5
Psychoanalysis	376	6.3	846	6.5	2.3
Mindfulness	5,498	92.6	11,981	92.3	2.2
	5,935	100.0	12,980	100.0	

*The percentages (%) are calculated with respect to the total number of tweets and likes respectively. CBT, Cognitive-Behavioral Therapy*.

Next, the interest generated by these tweets among social media users was investigated by analyzing the number of associated likes. In total, 12,980 likes were generated (Table 1). Interestingly, cognitive-behavioral therapy tweets showed the highest number of likes per tweet (Table 1).

### Content of the Tweets

Notably, different patterns of tweet distribution among the different content categories were found between the three psychotherapies (Table 2). The content that generated the highest frequency of tweets was that related to efficacy. Interestingly, all the tweets related to cognitive-behavioral therapy and half of those related to mindfulness included a positive consideration of their efficacy (Table 2). In contrast, none of the tweets related to psychoanalysis addressed efficacy.

**Table 2:** Number of tweets posted from 2009 to 2019 by 25 major US media outlets and retweets generated by their followers about psychotherapies according to different characteristics.

	**Efficacy**						**Specific condition**						**Link to a source included within the tweet**					
	**No mention**		**Yes**		**No**		**No mention**		**Physical health**		**Mental health**		**No link**		**Scientific**		**Non-scientific**	
	***N***	**%**	***N***	**%**	***N***	**%**	***N***	**%**	***N***	**%**	***N***	**%**	***N***	**%**	***N***	**%**	***N***	**%**
CBT	–	–	61	100	–	–	22	36.1	14	23.0	25	41.0	–	–	–	–	61	100
Psychoanalysis	376	100	–	–	–	–	375	99.7	–	–	1	0.3	–	–	–	–	376	100
Mindfulness	2,577	46.9	2,869	52.2	52	0.95	3,697	67.2	495	9.0	1,306	23.8	16	0.3	–	–	5,482	99.7
Total	2,953	49.8	2,930	49.4	52	0.9	4,094	69.0	509	8.6	1,332	22.4	16	0.3	–	–	5,919	99.7

*The percentages (%) are calculated with respect to the total number of tweets and retweets of each psychotherapy. CBT, Cognitive-Behavioral Therapy*.

The analysis of the content of those tweets related to whether specific aspects of physical and mental health were addressed also showed differences between the three types of psychotherapy. Nearly none of the psychoanalysis-related posts included references to either physical or mental conditions. In contrast, two-thirds of those related to cognitive-behavioral therapy included references to health, mainly focusing on mental aspects (Table 2).

The study of the inclusion of scientific or non-scientific sources within the tweets and retweets showed that the majority included a link to a non-scientific source. Finally, we found differences between the distribution of those tweets, including a reference to a celebrity, among the three different groups of psychotherapies (Table 2). More specifically, the highest percentage of tweets mentioning a celebrity were found in those posts related to psychoanalysis.

**Figure 1:**
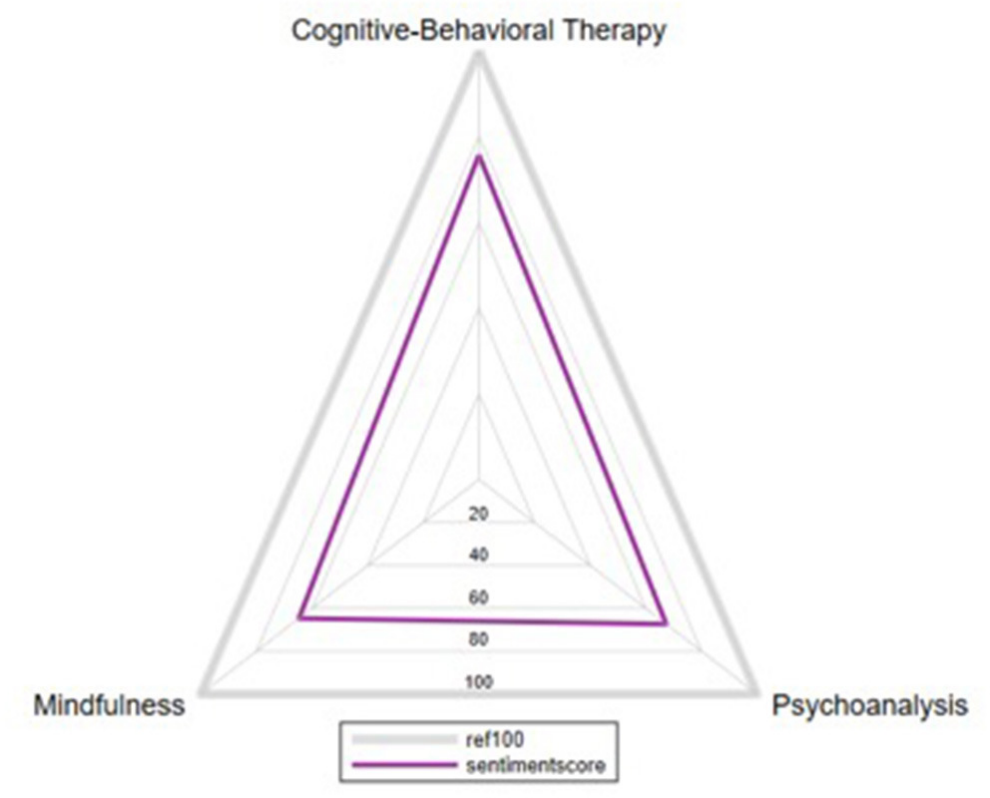
Sentiment analysis of each type of psychotherapy analyzed. The average score obtained by all the tweets with a certain hashtag determines the overall score of each food group (Cognitive-Behavioral Therapy, 75.69, Psychoanalysis, 67.3, Mindfulness, 64.7).

Regarding the sentiment analyses of the tweets (Figure 1), the three psychotherapies studied obtained a positive score between 60 and 80.

### Number of Mass Media Tweets and Follower's Retweets Throughout the Years

**Figure 2:**
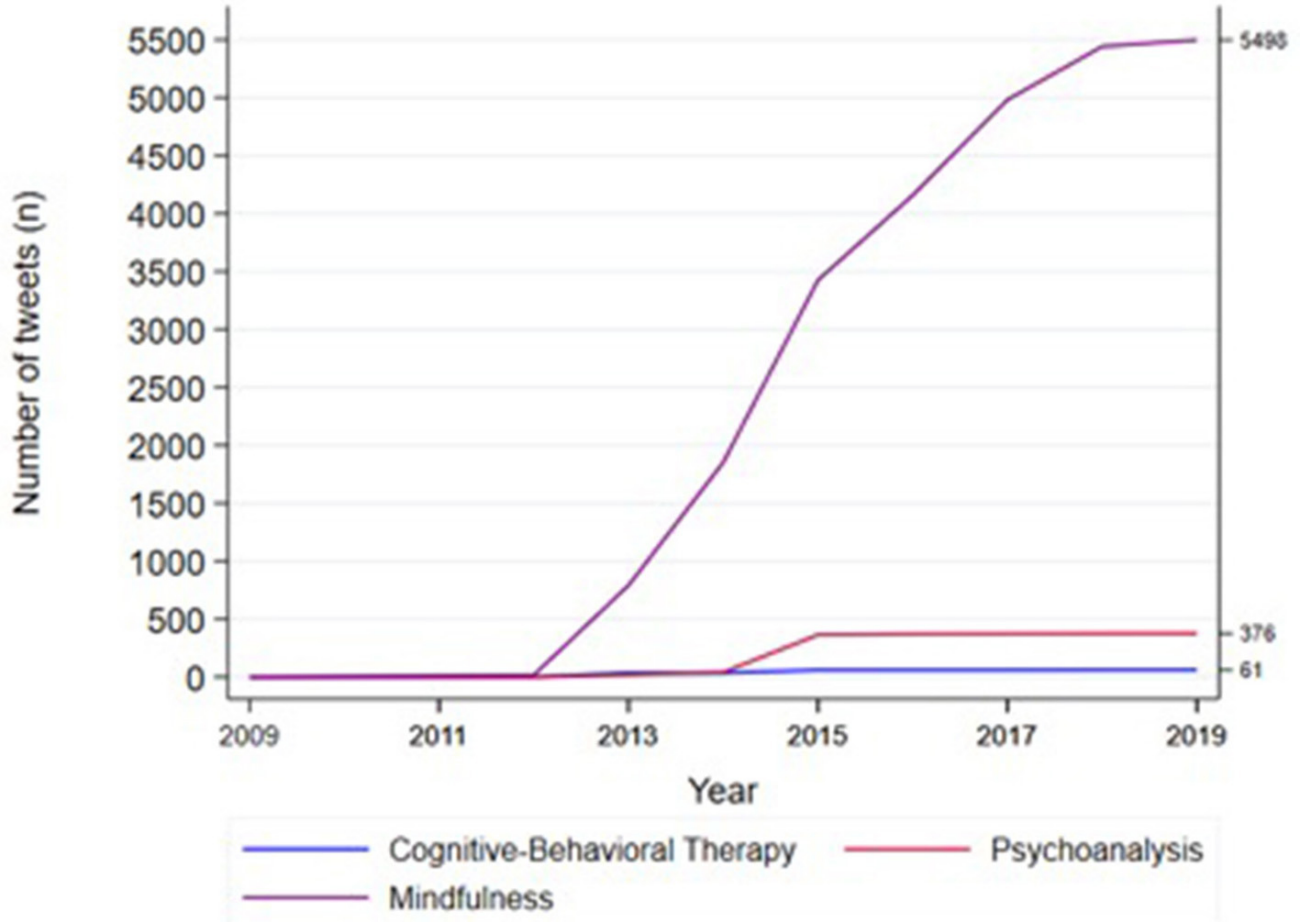
Time trend of all tweets posted by the 25 major US media outlets selected in the study and retweets generated by their followers between 2009 and 2019.

The evolution of the number of tweets related to psychotherapy posted by the 25 U.S. media outlets was assessed, as well as the retweets that were generated during the 11 years of the study (Figure 2). In this regard, a consistent number of generated tweets and retweets about psychoanalysis and cognitive-behavioral therapy was observed from 2014 onwards (Figure 2), while mindfulness generated tweets and retweets beginning in 2012, with a particular peak from 2013 to 2015 followed by a steady growth (Figure 2).

**Figure 3:**
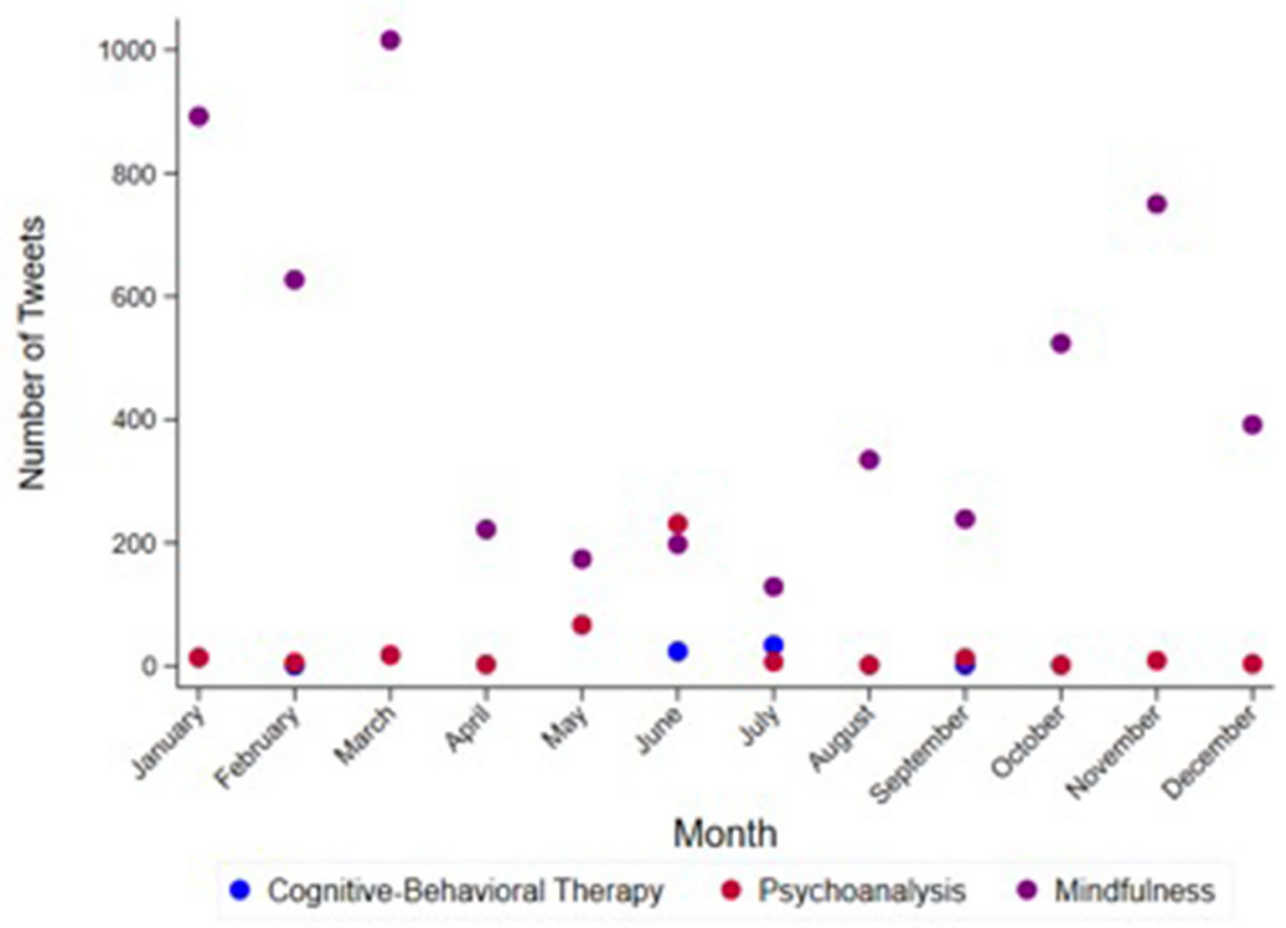
Monthly distribution of tweets posted by 25 US media outlets and the retweets generated by Twitter users between 2009 and 2019. The scatterplot represents all the tweets posted by the 25 major US media outlets selected in the study and the retweets generated by their followers. Each psychotherapy group is represented by a dot according to the following categories: Cognitive-Behavioral Therapy, Psychoanalysis and Mindfulness. Comparisons between months were not significant (*p*-value = 0.981). *P*-value by Kruskall-Wallis H test.

In addition, the number of tweets generated over months was investigated, without any temporal variability found in the frequency of those tweets related to psychotherapy (Figure 3). In particular, tweets referring to psychoanalysis increased in the month of June, cognitive-behavioral therapy tweets maintained a similar proportion throughout the year, and tweets referring to mindfulness peaked in the autumn and winter (Figure 3).

## Discussion

### Principal Findings

In this study, according to the tweets posted, it was found that the main U.S. media outlets and their Twitter followers were more interested in discussing mindfulness, while a very limited number of tweets were related to cognitive-behavioral therapy or psychoanalysis. Surprisingly, the other five psychotherapy modalities included in our study (computer-supported therapy, psychodynamic therapy, systemic therapy, acceptance and commitment therapy, and dialectical behavior therapy) did not generate any tweets. In terms of content, efficacy was the main focus of the tweets analyzed, with cognitive-behavioral therapy and mindfulness receiving positive appraisal in the majority of cases. However, this was not the case for psychonalysis.

Psychoanalysis still retains consideration in some sectors of psychology and psychiatry as a method to discover the meaning and motivation of behavior, especially the unconscious elements that might inform thoughts and feelings. However, it has been widely challenged for its poor therapeutic efficacy. In fact, within clinical guidelines, psychoanalysis is not included among recommended treatments for the most frequent psychiatric disorders. Thus, it is not surprising that its efficacy has not been discussed on Twitter. On the other hand, cognitive-behavioral therapy and mindfulness have been demonstrated to be effective for a wide range of psychological problems, as well as for reducing dysfunction in patients with medical conditions such as cancer or chronic pain (34, 35). Nonetheless, it is surprising how well regarded they are in terms of their efficacy, especially cognitive-behavioral therapy, which was considered an effective treatment in all related tweets. Interestingly, other treatments that have been shown to be effective in treating certain pathologies have not received as positive an assessment as cognitive-behavioral therapy has. For example, a previous study reported that liraglutide and semaglutide were rated as effective for treating obesity by 59.1 and 79.1% respectively from among the Twitter users discussing this topic (36). In another study that analyzed Twitter posts mentioning drugs used to treat ADHD, it was found that between 54.1 and 74.1% of the users who posted on this topic gave a positive assessment of their efficacy (37).

The data from our study shows that the number of tweets about psychotherapy sent by the 25 major U.S. outlets assessed over the past decade was unevenly distributed among the different psychotherapies, with the majority of tweets focusing on mindfulness. Nonetheless, in previous studies that have analyzed the interest of the media toward health issues, a similar pattern, but one less pronounced, has been detected. For instance, a study published in 2018 reviewed media posts on mental health from major U.S. media outlets for over a decade and found that half of the tweets discussed issues related to suicide or gender dysphoria (38). This same study also analyzed posts about physical illnesses, and found that almost half of the tweets were centered on breast cancer and HIV (38). Interestingly, a recently published study that also assessed posts from principal U.S. media outlets related to the Mediterranean diet and its components also observed that almost half of the tweets made reference to dairy products (39). Therefore, it seems that the media, when discussing a medical issue, tends to focus on certain aspects while ignoring others (33). This may explain why five of the eight psychotherapies included in our study have been omitted despite the fact that their use in clinical practice is widely accepted (40, 41). For example, dialectical behavior therapy has been proven to be one of the most effective therapies for reducing self-harm and suicidal thoughts in patients with borderline personality disorder, while systemic therapy has proven to be the most effective therapy for anorexia nervosa in adolescents, yet they did not generate any tweets (42, 43).

In addition to the aforementioned, the interests of media outlets and clinicians may differ for additional reasons. First, mindfulness is perhaps the most accessible psychotherapeutic modality to the general public since its use is not restricted to just psychiatrists and psychologists (44). To carry this therapy out does not require as much professional qualification as, for example, psychoanalysis, dialectical behavior therapy or acceptance and commitment therapy do. Secondly, mindfulness is also used to promote psychological well-being among people who are not diagnosed with a psychiatric disorder; therefore, the potential number of interested people is greater than with the rest of the other psychotherapies. In fact, its use is promoted among university students and in the workplace (45, 46). Third, the benefits of the other psychotherapeutic modalities take considerably more time to be observed than those of mindfulness, an aspect of treatment that is generally viewed as less appealing among patients and their family members. Fourth, unlike the other psychotherapies, mindfulness does not require family involvement, thus favoring its more widespread use. Finally, its application has been actively promoted in recent years, and even apps and podcasts have been designed to facilitate its use by the population (47). All these reasons may therefore explain why mindfulness is the psychotherapeutic modality that most captures the media's attention.

Moreover, we have identified a pronounced increase in the number of tweets generated on mindfulness that might reflect the increase in popularity and use that this technique has recently experienced (48). Interestingly, these mindfulness-related tweets did not include scientific references despite the fact that there is much scientific evidence on the therapy's health effects. However, media outlets should be concerned with issues of scientific origin because the inclusion of these types of links impacts the scientific appropriateness of the news (49). Lastly, the tone of tweet content posted by the major U.S. media outlets was analyzed, with data showing that these media outlets had a positive attitude toward the three psychotherapies. While it is extremely challenging to estimate the effect that these articles may have on health behaviors, media outlets do play an important role in generating opinion and it would therefore be beneficial to involve mental health professionals in these discussions to ensure scientific correctness when informing the population (50). For example, a recent study identified mass media as the main source for providing information on health affairs to the general public (51). Therefore, the media has the responsibility to offer truthful information that may be helpful to improve the quality of life and health of the population. Indeed, media outlets should also promote healthy behaviors and give voice to that research which also tends to interest readers (52). Posting links to scientific articles can expand readership to a wider audience. For example, three tweets about a Cochrane review increased visits to its webpage, and readers linking to the webpage via Twitter spent more time on the page than those arriving from other sources (53).

### Limitations

This study does have some limitations. First, since our data came exclusively from 25 selected national mainstream media Twitter accounts, we did not include data from local media accounts, which may also play a meaningful role in health issues and may contain a different set of priorities. Thus, it could be interesting to extend future research into a variety of other media types, including social media platforms such as Facebook and Instagram, in order to obtain a wider scope of how the topic of our research is viewed. Secondly, Twitter users are usually younger, have more education, and have higher incomes than the population at large, limiting the generalization of our results. Third, a more extensive list of keywords could have led to more comprehensive findings. Fourth, we have limited our analysis to tweets in English which may limit the ability to generalize the results since the non-English speaking population may have different interests or concerns in relation to psychotherapy. Finally, the content analysis process used has a degree of subjectivity that we overcame by having clinicians rate the tweets.

## Conclusions

To our knowledge, this project is the first to study the presence of psychotherapy in media outlets. Our results help to understand the role played by mass media with regards to favoring public awareness toward the importance of psychotherapy. U.S. media outlets have focused their interest on mindfulness, which may have contributed to the growing popularity in the past several years of this therapeutic modality. Although this study is focused on the U.S. media, these results provide relevant information which more than likely can be applicable to other countries.

## Data Availability Statement

The raw data supporting the conclusions of this article will be made available by the authors, without undue reservation.

## Author Contributions

MiA-M, MeA-M, and MM-G participated as principal contributors in the research design, manuscript writing, and submission. MO and CV participated in the content analysis and review. CF-L conducted and reported statistical analysis. RM-R participated in the review. MeA-M and MM-G contributed as supervisors of all the stages. All authors contributed to the article and approved the submitted version.

## Funding

This work was partially supported by grants from the Instituto de Salud Carlos III (PI18/01726) (Spain), the Programa de Actividades de I+D de la Comunidad de Madrid en Biomedicina (B2017/BMD-3804), Madrid (Spain), and Helekulani SL.

## Conflict of Interest

The authors declare that the research was conducted in the absence of any commercial or financial relationships that could be construed as a potential conflict of interest.

## Publisher's Note

All claims expressed in this article are solely those of the authors and do not necessarily represent those of their affiliated organizations, or those of the publisher, the editors and the reviewers. Any product that may be evaluated in this article, or claim that may be made by its manufacturer, is not guaranteed or endorsed by the publisher.

## References

[1] Jorm AF Patten SB Brugha TS Mojtabai R. Has increased provision of treatment reduced the prevalence of common mental disorders? Review of the evidence from four countries. World Psychiatry. (2017) 16:90–9. 10.1002/wps.20388 28127925 PMC5269479

[2] Ijaz S Davies P Williams CJ Kessler D Lewis G Wiles N. Psychological therapies for treatment-resistant depression in adults. Cochrane Database Syst Rev. (2018) 2018:CD010558. 10.1002/14651858.CD010558.pub2 29761488 PMC6494651

[3] Cuijpers P Sijbrandij M Koole SL Andersson G Beekman AT Reynolds CF. Adding psychotherapy to antidepressant medication in depression and anxiety disorders: a meta-analysis. World Psychiatry. (2014) 13:56–67. 10.1002/wps.20089 24497254 PMC3918025

[4] Cuijpers P Noma H Karyotaki E Vinkers CH Cipriani A Furukawa TA. network meta-analysis of the effects of psychotherapies, pharmacotherapies and their combination in the treatment of adult depression. World Psychiatry. (2020) 19:92–107. 10.1002/wps.20701 31922679 PMC6953550

[5] Swift JK Callahan JL Cooper M Parkin SR. The impact of accommodating client preference in psychotherapy: a meta-analysis. J Clin Psychol. (2018) 74:1924–37. 10.1002/jclp.22680 30091140

[6] Tompkins KA Swift JK Callahan JL. Working with clients by incorporating their preferences. Psychotherapy. (2013) 50:279–83. 10.1037/a0032031 24000835

[7] Norcross JC Wampold BE. A new therapy for each patient: evidence-based relationships and responsiveness. J Clin Psychol. (2018) 74:1889–906. 10.1002/jclp.22678 30334258

[8] Falkenström F. Markowitz, JC, Jonker H, Philips B, Holmqvist R. Can psychotherapists function as their own controls? J Clin Psychiatry. (2013) 74:482–91. 10.4088/JCP.12r07848 23146326 PMC3683365

[9] Greenberg RP Constantino MJ Bruce N. Are patient expectations still relevant for psychotherapy process and outcome? Clin Psychol Rev. (2006) 26:657–78. 10.1016/j.cpr.2005.03.002 15908088

[10] Constantino MJ Arnkoff DB Glass CR Ametrano RM Smith JZ. Expectations. J Clin Psychol. (2011) 67:184–92. 10.1002/jclp.20754 21128304

[11] Constantino MJ Vîslă A Coyne AE Boswell JF. A meta-analysis of the association between patients' early treatment outcome expectation and their posttreatment outcomes. Psychotherapy. (2018) 55:473–85. 10.1037/pst0000169 30335459

[12] Ducrot P Montagni I Nguyen Thanh V Serry A-J Richard J-B. Evolution of online health-related information seeking in France from 2010 to 2017: results from nationally representative surveys. J Med Internet Res. (2021) 23:e18799. 10.2196/18799 33851927 PMC8082381

[13] Fassier P Chhim A-S Andreeva VA Hercberg S Latino-Martel P Pouchieu C. Seeking health- and nutrition-related information on the Internet in a large population of French adults: results of the NutriNet-Santé study. Br J Nutr. (2016) 115:2039–46. 10.1017/S0007114516001355 27081008

[14] Pew, Research Center. Social Media Use in 2021. Available online at: https://www.pewresearch.org/internet/2021/04/07/social-media-use-in-2021/ (accessed February 7, 2022).

[15] Pew, Research Center. News Use Across Social Media Platforms in 2020. Available online at: https://www.pewresearch.org/journalism/2021/01/12/news-use-across-social-media-platforms-in-2020/ (accessed February 7, 2022).

[16] Pew, Research Center. How Americans Tweet About the News. Available online at: https://www.pewresearch.org/fact-tank/2021/12/14/how-americans-tweet-about-the-news/ (accessed February 7, 2022).

[17] Metwally O Blumberg S Ladabaum U Sinha SR. Using social media to characterize public sentiment toward medical interventions commonly used for cancer screening: an observational study. J Med Internet Res. (2017) 19:e200. 10.2196/jmir.7485 28592395 PMC5480009

[18] Allem J-P Escobedo P Dharmapuri L. Cannabis surveillance with twitter data: emerging topics and social bots. Am J Public Health. (2020) 110:357–62. 10.2105/AJPH.2019.305461 31855475 PMC7002948

[19] Nguyen QC McCullough M Meng H-W Paul D Li D Kath S. Geotagged US tweets as predictors of county-level health outcomes, 2015–2016. Am J Public Health. (2017) 107:1776–82. 10.2105/AJPH.2017.303993 28933925 PMC5637661

[20] Green Lauridsen M Kälvemark Sporrong S. How does media coverage effect the consumption of antidepressants? A study of the media coverage of antidepressants in Danish online newspapers 2010–2011. Res Soc Adm Pharm. (2018) 14:638–644. 10.1016/j.sapharm.2017.07.011 28811152

[21] Saha K Torous J Caine ED De Choudhury M. Psychosocial effects of the COVID-19 pandemic: large-scale quasi-experimental study on social media. J Med Internet Res. (2020) 22:e22600. 10.2196/22600 33156805 PMC7690250

[22] Pereira-Sanchez V Alvarez-Mon MA. Asunsolo del Barco A, Alvarez-Mon M, Teo A. Exploring the extent of the hikikomori phenomenon on twitter: mixed methods study of western language tweets. J Med Internet Res. (2019) 21:e14167. 10.2196/14167 31144665 PMC6658314

[23] Berry N Lobban F Belousov M Emsley R Nenadic G Bucci S. #WhyWeTweetMH: understanding why people use twitter to discuss mental health problems. J Med Internet Res. (2017) 19:e107. 10.2196/jmir.6173 28381392 PMC5399219

[24] Saha K Torous J Ernala SK Rizuto C Stafford A De Choudhury M. computational study of mental health awareness campaigns on social media. Transl Behav Med. (2019) 9:1197–207. 10.1093/tbm/ibz028 30834942 PMC6875652

[25] Saha K Torous J Kiciman E De Choudhury M. Understanding side effects of antidepressants: large-scale longitudinal study on social media data. JMIR Ment Heal. (2021) 8:e26589. 10.2196/26589 33739296 PMC8077932

[26] Olmstead K Mitchell A Rosenstiel T. Navigating News Online: Where People Go, How They Get There and What Lures Them Away. Washington, DC: Pew Research Center (2011).

[27] Morstatter F Pfeffer J Liu H Carley KM. Is the Sample Good Enough? Comparing Data From Twitter's Streaming API With Twitter's Firehose. (2013).

[28] Alvarez-Mon MA Donat-Vargas C Santoma-Vilaclara J de Anta L Goena J Sanchez-Bayona R. Assessment of antipsychotic medications on social media: machine learning study. Front psychiatry. (2021) 12:737684. 10.3389/fpsyt.2021.737684 34867531 PMC8637121

[29] de Anta L Alvarez-Mon MA Ortega MA Salazar C Donat-Vargas C Santoma-Vilaclara J. Areas of interest and social consideration of antidepressants on english tweets: a natural language processing classification study. J Pers Med. (2022) 12:155. 10.3390/jpm12020155 35207644 PMC8879287

[30] Pereira-Sanchez V Alvarez-Mon MA Horinouchi T Kawagishi R Tan MPJ Hooker ER. Examining tweet content and engagement of users with tweets about Hikikomori in Japanese: mixed methods study of social withdrawal. J Med Internet Res. (2022) 24:e31175. 10.2196/31175 35014971 PMC8925292

[31] Alvarez-Mon MA Llavero-Valero M Sánchez-Bayona R Pereira-Sanchez V Vallejo-Valdivielso M Monserrat J. Areas of interest and stigmatic attitudes of the general public in five relevant medical conditions: thematic and quantitative analysis using twitter. J Med Internet Res. (2019) 21:e14110. 10.2196/14110 31140438 PMC6658306

[32] Viguria I Alvarez-Mon MA Llavero-Valero M. Asunsolo del Barco A, Ortuño F, Alvarez-Mon M. Eating disorder awareness campaigns: thematic and quantitative analysis using twitter. J Med Internet Res. (2020) 22:e17626. 10.2196/17626 32673225 PMC7388051

[33] Alvarez-Mon MA Donat-Vargas C Llavero-Valero M Gea A Alvarez-Mon M Martinez-Gonzalez MA. Analysis of media outlets on women's health: thematic and quantitative analyses using twitter. Front Public Heal. (2021) 9:644284. 10.3389/fpubh.2021.644284 34136450 PMC8200480

[34] Jelvehzadeh F Dogaheh ER Bernstein C Shakiba S Ranjbar H. The effect of a group cognitive behavioral therapy on the quality of life and emotional disturbance of women with breast cancer. Support Care Cancer. (2022) 30:305–12. 10.1007/s00520-021-06421-4 34278530

[35] Pardos-Gascón EM Narambuena L Leal-Costa C Ramos-Morcillo AJ Ruzafa-Martínez M. van-der Hofstadt Román CJ. Psychological therapy in chronic pain: differential efficacy between mindfulness-based cognitive therapy and cognitive behavioral therapy. J Clin Med. (2021) 10:3544. 10.3390/jcm10163544 34441842 PMC8397134

[36] Alvarez-Mon MA Llavero-Valero M Asunsolo del Barco A Zaragozá C Ortega MA Lahera G. Areas of interest and attitudes toward antiobesity drugs: thematic and quantitative analysis using twitter. J Med Internet Res. (2021) 23:e24336. 10.2196/24336 34698653 PMC8579215

[37] Alvarez-Mon MA de Anta L Llavero-Valero M Lahera G Ortega MA Soutullo C. Areas of interest and attitudes towards the pharmacological treatment of attention deficit hyperactivity disorder: thematic and quantitative analysis using twitter. J Clin Med. (2021) 10:2668. 10.3390/jcm10122668 34204353 PMC8235344

[38] Alvarez-Mon MA Asunsolo del Barco A Lahera G Quintero J Ferre F Pereira-Sanchez V. Increasing interest of mass communication media and the general public in the distribution of tweets about mental disorders: observational. Study J Med Internet Res. (2018) 20:e205. 10.2196/jmir.9582 29807880 PMC5996178

[39] Alvarez-Mon MA Fernandez-Lazaro CI Llavero-Valero M Alvarez-Mon M Mora S Martínez-González MA. Mediterranean diet social network impact along 11 years in the major US media outlets: thematic and quantitative analysis using twitter. Int J Environ Res Public Health. (2022) 19:784. 10.3390/ijerph19020784 35055605 PMC8775755

[40] Eccleston C Fisher E Law E Bartlett J Palermo TM. Psychological interventions for parents of children and adolescents with chronic illness. Cochrane Database Syst Rev. (2015) 4:CD009660. 10.1002/14651858.CD009660.pub3 25874881 PMC4838404

[41] van Agteren J Iasiello M Lo L Bartholomaeus J Kopsaftis Z Carey M. systematic review and meta-analysis of psychological interventions to improve mental wellbeing. Nat Hum Behav. (2021) 5:631–52. 10.1038/s41562-021-01093-w 33875837

[42] Linehan MM Comtois KA Murray AM Brown MZ Gallop RJ Heard HL. Two-year randomized controlled trial and follow-up of dialectical behavior therapy vs. therapy by experts for suicidal behaviors and borderline personality disorder. Arch Gen Psychiatry. (2006) 63:757–66. 10.1001/archpsyc.63.7.757 16818865

[43] Treasure J Parker S Oyeleye O Harrison A. The value of including families in the treatment of anorexia nervosa. Eur Eat Disord Rev. (2021) 29:393–401. 10.1002/erv.2816 33351987 PMC8246805

[44] Taylor H Strauss C Cavanagh K. Can a little bit of mindfulness do you good? A systematic review and meta-analyses of unguided mindfulness-based self-help interventions. Clin Psychol Rev. (2021) 89:102078. 10.1016/j.cpr.2021.102078 34537665

[45] Regehr C Glancy D Pitts A. Interventions to reduce stress in university students: a review and meta-analysis. J Affect Disord. (2013) 148:1–11. 10.1016/j.jad.2012.11.026 23246209

[46] Sekhar P Tee QX Ashraf G Trinh D Shachar J Jiang A. Mindfulness-based psychological interventions for improving mental well-being in medical students and junior doctors. Cochrane Database Syst Rev. (2021) 2021:CD013740. 10.1002/14651858.CD013740.pub2 34890044 PMC8664003

[47] Gál É Stefan S Cristea IA. The efficacy of mindfulness meditation apps in enhancing users' well-being and mental health related outcomes: a meta-analysis of randomized controlled trials. J Affect Disord. (2021) 279:131–42. 10.1016/j.jad.2020.09.134 33049431

[48] Wang Y Liao L Lin X Sun Y Wang N Wang J. Bibliometric and visualization analysis of mindfulness and meditation research from 1900 to 2021. Int J Environ Res Public Health. (2021) 18:13150. 10.3390/ijerph182413150 34948760 PMC8701075

[49] Schwartz LM Woloshin S Andrews A Stukel TA. Influence of medical journal press releases on the quality of associated newspaper coverage: retrospective cohort study. BMJ. (2012) 344:d8164–d8164. 10.1136/bmj.d8164 22286507 PMC3267473

[50] Schwartz LM Woloshin S. News media coverage of screening mammography for women in their 40s and tamoxifen for primary prevention of breast cancer. JAMA. (2002) 287:3136–42. 10.1001/jama.287.23.3136 12069679

[51] Falcone R Sapienza A. How COVID-19 changed the information needs of Italian citizens. Int J Environ Res Public Health. (2020) 17:6988. 10.3390/ijerph17196988 32987914 PMC7579097

[52] Stryker JE. Reporting medical information: effects of press releases and newsworthiness on medical journal articles' visibility in the news media. Prev Med. (2002) 35:519–30. 10.1006/pmed.2002.1102 12431901

[53] Jayaram M Moran L Adams C. Twittering on about mental health: is it worth the effort? Evid Based Ment Health. (2017) 20:1–3. 10.1136/eb-2016-102580 28100506 PMC10688418

